# A phase I trial of the trifunctional anti Her2 × anti CD3 antibody ertumaxomab in patients with advanced solid tumors

**DOI:** 10.1186/s12885-016-2449-0

**Published:** 2016-07-07

**Authors:** N. Haense, A. Atmaca, C. Pauligk, K. Steinmetz, F. Marmé, G. M. Haag, M. Rieger, O. G. Ottmann, P. Ruf, H. Lindhofer, S.-E. Al-Batran

**Affiliations:** Institute of clinical research (IKF) at Krankenhaus Nordwest, UCT-University Cancer Center, Steinbacher Hohl 2-26, 60488 Frankfurt am Main, Germany; Department of Hematology and Oncology, Krankenhaus Nordwest, UCT-University Cancer Center, Steinbacher Hohl 2-26, 60488 Frankfurt am Main, Germany; Department of Medical Oncology, National Center for Tumor Diseases, University Hospital Heidelberg, Im Neuenheimer Feld 460, 69120 Heidelberg, Germany; Onkologische Schwerpunktpraxis, Eschollbrücker Str. 26, 64295 Darmstadt, Germany; Department of Medicine, Hematology and Oncology, Johann Wolfgang Goethe University, Frankfurt, Germany; TRION Research GmbH, Am Klopferspitz 19, 82152 Martinsried, Germany

**Keywords:** Ertumaxomab, Her2/neu, Advanced cancer, Dose limiting toxicity, Dose escalation, Maximum tolerated dose

## Abstract

**Background:**

Ertumaxomab (ertu) is a bispecific, trifunctional antibody targeting Her2/neu, CD3 and the Fcγ-receptors I, IIa, and III forming a tri-cell complex between tumor cell, T cell and accessory cells.

**Methods:**

Patients (pts) with Her2/neu (1+/SISH positive, 2+ and 3+) expressing tumors progressing after standard therapy were treated to investigate safety, tolerability and preliminary efficacy. In this study, ertu was applied i.v. in 2 cycles following a predefined dose escalating scheme. Each cycle consisted of five ascending doses (10–500 μg) applied weekly within 28 days with a 21 day treatment-free interval. If 2 pts experienced a dose limiting toxicity (DLT) at a given dose level, the maximum tolerated dose (MTD) had been exceeded.

**Results:**

Fourteen heavily pretreated pts (e.g. breast, rectal, gastric cancer) were enrolled in the four main cohorts. Three (21 %) pts had to be replaced. Two serious adverse events (SAE) with possible relation to the investigational drug were seen, both fully reversible. A DLT was not detected. Consequently, the MTD could not be determined. All adverse events (AE) were transient and completely reversible. Most frequent AEs were fatigue (14/14), pain (13/14), cephalgia (12/14), chills (11/14), nausea (8/14), fever (7/14), emesis (7/14) and diarrhea (5/14). Single doses up to 300 μg were well tolerated (total dose up to 800 μg per cycle). We observed one partial remission and two disease stabilizations after first treatment cycle.

**Conclusions:**

Single doses up to 300 μg could be safely administered in an escalating dose scheme. Immunological responses and clinical activity warrant further evaluation in patients with Her2 over expressing tumors.

**Trial registration:**

EudraCT number: 2011-003201-14; ClinicalTrials.gov identifier: NCT01569412

## Background

The most known member of the epidermal growth receptor family, Her2/neu, is frequently found to be overexpressed in various types of cancers like breast cancer, gastric cancer, lung cancer and ovarian cancer. An increased Her2/neu expression results in a more aggressive tumor behavior. Thus, many studies have indicated that Her2/neu overexpression is associated with a poor prognosis and with a significantly shorter overall survival rate and time to relapse for patients with Her2/neu expressing tumors [1–3].

The Her2/neu (c-ErbB-2) proto-oncogene encodes an 185kD trans-membrane glycoprotein that takes action as a tyrosine kinase receptor [3]. As a tyrosine kinase receptor, Her2/neu participates in an interactive network of receptor interactions resulting in complex signaling pathways to control and regulate cell growth, migration, differentiation and death [3–5].

An overexpression of Her2/neu, often caused by amplification of the c-ErbB-2 gene, leads to enhanced tyrosine phosphorylation activity and therefore to increased cell proliferation and metastatic transformation in tumors [5, 6]. Thus, the Her2/neu receptor serves as an effective target for antineoplastic agents [6].

Bispecific antibodies are a new and promising approach for immunologic treatment of cancer cells. Therefore, a simultaneous and powerful activation of effector cells such as T cells, NK cells and dendritic cells is preferable. However, the bispecific antibodies generated so far normally activate only a single class of effector cells resulting in an insufficient immunologic attack against tumor cells [7]. Triomab® antibodies represent a new class of bispecific, hybrid-hybridoma derived antibodies configured of two potent subclasses of mouse IgG2a and a rat IgG2b chain. As a welcoming result of this structure, triomab® antibodies possess three different binding sides: a tumor-associated antigen (TAA)-specific binding arm, a second arm specific for CD3 expressed on T cells, and a chimeric mouse IgG2a x rat IgG2b Fc region that recognizes Fcγ receptors type I, IIa and III present on accessory cells such as macrophages, dendritic cells or NK cells [8–10].

**Fig. 1:**
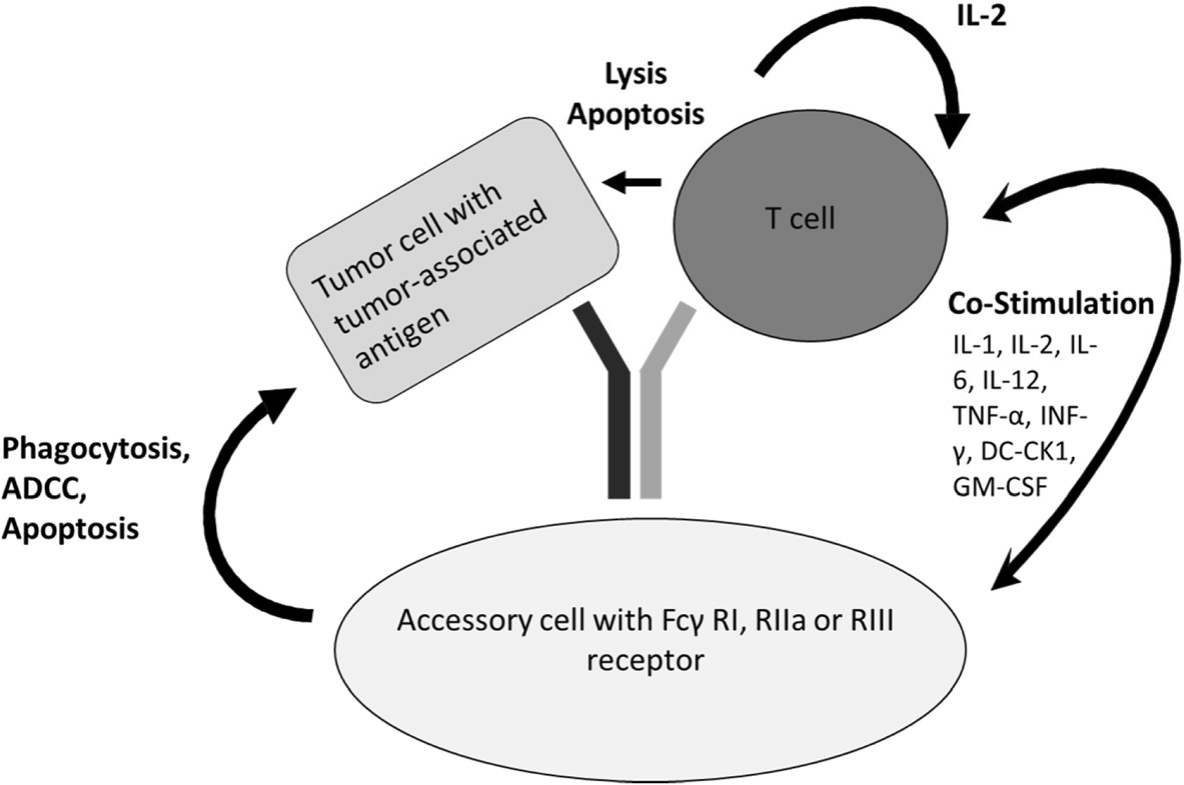
Mode of action of a triomab antibody. The trifunctional antibody unites tumor cell, T cell and accessory cells to form a tri-cell complex to induce tumor cell destruction and phagocytosis. Abbreviations: ADCC, antibody dependent cellular cytotoxicity; DC, dendritic cell; DC-CK1, dendritic cell cytokine 1; IL, interleukin; LFA, leukocyte function associated antigen; NK, natural killer cell; TNF-α, tumor necrosis factor alpha; INF-γ, interferon gamma; GM-CSF, granulocyte macrophage colony-stimulating factor. Modified^10,12^

Here, we report data on a new immunotherapeutic agent, ertumaxomab (ATC code L01XC). Ertumaxomab is a bispecific, trifunctional antibody that binds to Her2/neu as its tumor-associated antigen, to CD3 and to Fcγ forming a tri-cell complex between tumor cell, T cell and accessory cells (Fig. 1). As a result, various immunologic mechanisms are induced to initiate a polyclonal humoral and cellular immune response to destroy Her2/neu expressing tumor cells:

a) Mediation of T cell activation by CD3 binding results in tumor cell killing by e.g. cytokine release and release of lytic enzymes such as perforins [7, 11]. b) T cells receive a second activating signal by release of stimulating cytokines (e.g. IL-2) and a crosstalk between costimulatory molecules now expressed on T cells and accessory cells [7, 12]. c) Necrotic or apoptotic tumor particles are phagocytized by Fcγ positive cells such as macrophages resulting in uptake, processing and presentation of these tumor particles. Consequently, an anti-tumor immunization against Her2/neu and other unknown tumor-associated antigens is induced resulting in the generation of cytotoxic T-cells and tumor-specific antibody producing plasma cells. Finally, the mode of action of triomab® antibodies, e.g. ertumaxomab, develops a protective longterm anti-tumor immunity [9, 13, 14].

This effective attack of various immunologic cells activating complex immunologic mechanisms leads to significant tumor cell elimination. Heiss et al. demonstrated that patients with malignant ascites gain a clear clinical benefit when treated i.p. with the trifunctional anti-EpCAM x anti-CD3 antibody catumaxomab [15].

Recently, Kiewe et al. published promising data for the trifunctional anti-Her2/neu x anti-CD3 antibody ertumaxomab. In a phase I trial patients with metastatic breast cancer were enrolled and treated with the trifunctional antibody in a dose escalating scheme. A clinical response to ertumaxomab treatment was seen in five out of fifteen patients [12]. These results are encouraging and indicate antitumor efficacy. Also, the finding that ertumaxomab has a different mode of action compared with trastuzumab, a monoclonal anti-Her2/neu antibody [16], strengthened the idea of investigating this triomab® antibody in a second phase I study enrolling patients with Her2/neu (IHC 1+/ISH positive, 2+, 3+) expressing solid tumors.

A phase I trial was designed to investigate the safety, tolerability and preliminary efficacy of ertumaxomab in patients with solid tumors progressing after standard therapy. In order to increase the amount of applied doses and to reduce toxicity compared to a previous study in breast cancer [12], we used a modified dosing schedule with small dose escalation steps and five consecutive, weekly administrations.

## Methods

### Study design

The primary objective in this single center phase I trial was the evaluation of safety and tolerability of the trifunctional antibody ertumaxomab in patients with Her2/neu expressing solid tumors in order to determine the maximum tolerated dose (MTD) and to establish a recommended dose (RD) for further investigations. Secondary endpoints were antitumor activity (disease control rate) and the measurement of immunological response (anti-drug antibodies [HAMA], humoral immune responses [Anti-EpCam- and anti-Her2/neu antibodies], lymphocyte cell count).

The study (EudraCT number: 2011-003201-14; ClinicalTrials.gov identifier: NCT01569412) was conducted according to the principles of the International Conference of Harmonization-Good Clinical Practice and approved by the institutional ethics committee. All relevant authorities were notified according to German drug law.

### Patient eligibility

Patients were eligible if they had histologically confirmed solid Her2/neu positive (1+/ISH positive, 2+ and 3+) tumors, no available standard treatment, measurable disease according to RECIST 1.1., disease progression during or after standard therapy, age >18 years, ECOG performance status 0–2, adequate hematological, liver, kidney and cardiac function (LVEF >50 %). Unlike common criteria for anti-Her2/neu strategies, patients with score 2+ were eligible regardless of their ISH results, as “immunotherapy effects” were expected also in patients with moderate Her2/neu overexpression.

Patients were excluded if they had known hypersensitivity to murine proteins or other components of the drug, any concurrent chemotherapy, radiotherapy, hormone therapy, immunotherapy or treatment with any investigational drug within 2 weeks prior to study entry, a documented autoimmune disease, HIV, HBV, HCV, acute or chronic infections or other concurrent non-malignant co-morbidities, ≥5 preceding chemotherapy lines (to exclude potentially immunocompromised patients), prior diagnosis of any other uncured malignancy, any documented evidence of symptomatic brain or central nervous system metastases or abnormal organ or bone marrow function.

All patients signed an informed consent form before participating in the trial.

### Treatment plan and dose escalation

**Fig. 2:**
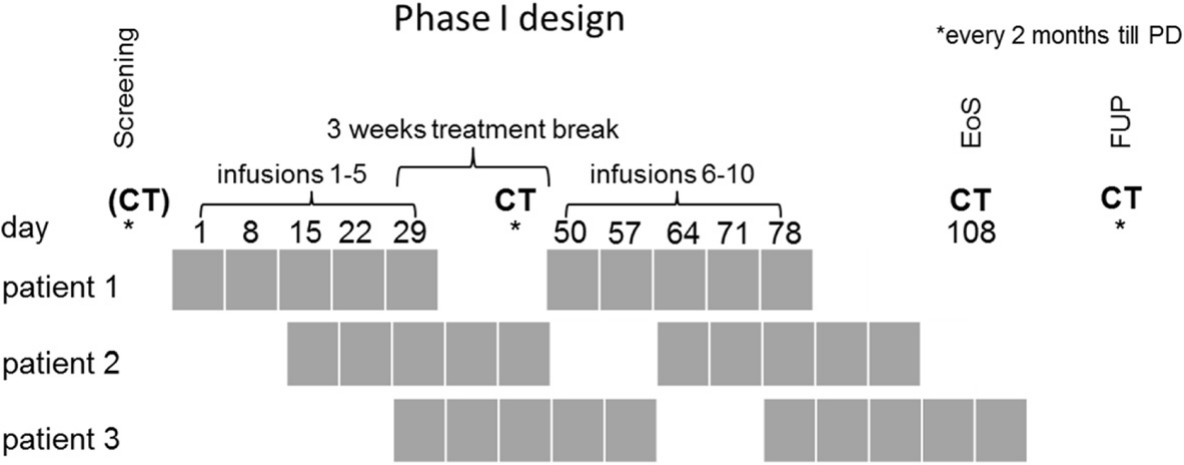
Study design. Abbreviations: CT, computed tomography; PD, progressive disease; EoS, End of Study; FUP, follow up

In this investigator driven, open label, uncontrolled trial the administration of ertumaxomab followed a predefined dose escalation scheme (Table 1) consisting of two dose-identical cycles with five ascending doses (10 μg to 500 μg) per cycle. In order to avoid cytokine release related symptoms associated with application of higher doses in treatment naïve patients, an intraindividual dose escalating schedule in each cohort was chosen, starting with low doses. Patients were treated once weekly from day 1 to 28 (i.e. one cycle) followed by a treatment-free interval of 21 days in-between (Fig. 2). The dose escalation into the next dose level occurred when three patients had received all five administrations of the first cycle without experiencing a dose limiting toxicity (DLT). If one patient had shown a DLT, additional three patients had to be enrolled and treated at the same dose level before proceeding into next dose level. If a DLT was seen in two patients at a given dose level, the MTD had been exceeded. Dose levels 1, 5, 9 and 13 were the main cohorts (in bold letters). The intermediate cohorts served to identify the MTD if ≥ 2 DLTs occurred in a main cohort (Table 1).

**Table 1:** Dose escalation scheme

Dose level	Escalation scheme [μg]	No. of pts
**1** ^**a**^	**10-50-100-100-100**	**3–6**
2	10-50-100-100-150	3–6
3	10-50-100-150-150	3–6
4	20-50-100-150-150	3–6
**5** ^**a**^	**20-50-100-150-200**	**3–6**
6	20-50-100-150-250	3–6
7	20-50-100-200-200	3–6
8	50-100-150-200-200	3–6
**9** ^**a**^	**20-50-100-200-300**	**3–6**
10	20-50-100-200-350	3–6
11	50-100-100-150-200	3–6
12	50-100-150-200-200	3–6
**13** ^**a**^	**50-100-150-200-300**	**3–6**
14	50-100-150-300-300	3–6
15	50-150-150-300-300	3–6
16	50-150-300-400-500	3–6

*Abbreviations* **:** *Pts* patients

^a^Main cohort

### Drug administration

Ertumaxomab was manufactured by TRION Pharma GmbH, Munich, under good manufacturing practice conditions and approved by the local authority. The antibody was supplied by TRION Pharma as a sterile, clear, colorless, preservative-free concentrate in prefilled syringes. The syringes contained 10 μg or 50 μg of antibody and had to be dissolved in 0.9 % sodium chloride solution to a total volume of 10 ml or 50 ml. Ertumaxomab was administered as an i.v. infusion with a constant rate over 3 h. Afterwards, 600 mg of Ibuprofen were given orally to allay possible side effects of the immunologic therapy. Patients remained under hospital care and surveillance for 24 h after start of the infusion.

### Toxicity assessment

Adverse Events (AE) were assessed at every visit by clinical examination and laboratory tests and graded according to the National Cancer Institute Common Toxicity Criteria (CTC), version 4.0. Respectively, patients were monitored frequently during their stay at the hospital and at the scheduled follow-up visits: medical examinations including vital signs, general physical examinations, electrocardiography and echocardiography were performed for safety before or during drug administration. Laboratory safety parameters including blood chemistry and hematology were measured before and 24 h after every infusion.

DLTs were predefined in the study protocol as any grade ≥3 drug related non-hematological toxicity except events that were not optimally treated with standard medication >3 days of duration, any irreversible grade ≥3 infusion-related reaction (defined as allergic reaction, fever, pain, bronchospasm, wheezing or hypoxia, occurring during or within 24 h after completing an infusion and resolving with a reduced infusion rate, supportive care and/or the administration of corticosteroids); any grade ≥3 elevation of liver enzymes that was not declining within 7 days after drug administration; any grade ≥4 toxicity, which have not been mentioned above as well as any grade ≥3 event considered to be a DLT by the investigator. Moreover, fatigue (CTC grade ≥3) lasting less than 14 days and isolated laboratory abnormalities grade ≥3 that resolved to baseline or CTC grade 2 within 7 days without clinical sequelae or need for therapeutic intervention were not considered as a DLT.

### Efficacy measurement

Tumor assessments according to response evaluation criteria in solid tumors (RECIST) were planned for the screening, the treatment-free interval before the second cycle (day 42–49) and for the end of study (day 108) as well as for follow up visits.

### Immunological analyses

As ertumaxomab is an antibody derived from mouse and rat IgG, it has the potential of immunogenicity when administered to humans. Thus, patient plasma samples for the determination of human anti-murine antibodies (HAMAs) were collected before the first cycle, before the second cycle and at the end of study. Testing for HAMAs was performed using the Medac test (Medac, Hamburg).

Also, humoral immune responses against tumor-associated antigens e.g. anti-EpCam- and anti-Her2/neu antibodies were measured during the treatment. For the detection of anti-EpCam antibodies, a bridging ELISA format was applied, anti-Her2 antibodies were measured by immobilization of recombinant Her2 (Bender Med Systems) to the plate surface and using Herceptin as a calibrator. These assays provided information about potential vaccination effects against various tumor-associated antigens evolving during ertumaxomab treatment.

Lymphocyte subsets were analyzed from peripheral blood samples obtained before each infusion and 24 h after each infusion. Percentage and absolute counts of the different lymphocyte populations were determined using the Becton Dickinson (BD) Multitest IMK kit.

### Statistics

This study was exploratory and not powered to address any pre-defined hypotheses. The safety and efficacy analysis was performed on every patient who received at least one infusion of ertumaxomab. Also, safety and efficacy were analyzed by appropriate descriptive statistics. All other endpoints were summarized descriptively.

## Results

### Patient characteristics

Out of 14 enrolled patients, three patients received only two administrations of the investigational antibody ertumaxomab and had to be replaced. Reasons for discontinuing the trial were allergic reaction, death and liver failure all classified as non DLT (e.g. disease related). Therefore, eleven patients were treated with ertumaxomab according to protocol. They all completed the first cycle, three of them continued with the second cycle. The patient characteristics are outlined in detail in Table 2.

**Table 2:** Patient characteristics

Patient characteristics	No. of patients (%) *n* = 14
Sex Male Female	4 (28.6) 10 (71.4)
Age Median age, years (range)	54,5 (35–72)
ECOG performance status O 1 2	8 (57.1) 5 (35.7) 1 (7.1)
Primary tumor location Breast Rectum Stomach Others^a^	5 (35.7) 3 (21.4) 3 (21.4) 3 (21.4)
No. of organs involved (primary excluded) 1 2 3 ≥4	5 (35.7) 3 (21.4) 2 (14.3) 4 (28.6)
Organs involved (primary tumor excluded) Lymph nodes Lung Liver Abdominal Wall Bone Others^b^	7 (50.0) 5 (35.7) 3 (21.4) 3 (21.4) 3 (21.4) 10 (71.4)
Her2 status IHC 2+/ISH+ IHC 3+	3 (21.4) 11 (78.6)

*Abbreviations*: *ECOG* Eastern Cooperative Oncology Group, *IHC* immunohistochemistry, *ISH* in-situ hybridisation

^a^Others: head and neck (2 pts), pancreas (1 pt)

^b^Others: adrenal gland, spleen, bladder, lymphangiosis carcinomatosa, pleura, peritoneum, chest wall, brain, skin

### Dose escalation

Fourteen patients were enrolled in four main cohorts with different dose levels: cohort 1 (10-50-100-100-100 μg), three patients; cohort 5 (20-50-100-150-200 μg), four patients; cohort 9 (20-50-100-200-300 μg), three patients; cohort 13 (50-100-150-200-300 μg); four patients. In this dose escalation scheme, single doses up to 300 μg were well tolerated. The received doses ranged from 10 μg to 300 μg as a single application and from 360 μg to 800 μg in total per cycle. The dose escalation proceeded into the 13th dose level (50-100-150-200-300 μg) without showing a DLT. Consequently, in this scheme the MTD is not reached and a RD not found so far.

### Safety

Out of all serious adverse events (SAEs) that occurred during the trial (*n* = 12), only two were possibly or certainly related to the administration of the investigated antibody ertumaxomab and thus classified as SAR (serious adverse reaction): one patient (#5) experienced an allergic reaction after the second application (50 μg). Her main symptom was dyspnea with pain in the upper abdomen. The other SAR emerged in patient #14 after his second infusion with 100 μg of ertumaxomab. He developed a fever (CTC grade 1) which was accompanied by other symptoms as chills, abdominal pain, nausea and vomiting leading to unplanned hospitalization. Both SARs were fully reversible.

All patients showed treatment-related toxicities. Most frequent adverse events (AEs) were fatigue (14/14 patients, 100 %), (tumor-) pain (13/14 patients, 93 %), cephalgia (12/14 patients, 86 %), chills (11/14 patients, 79 %), nausea (8/14 patients, 62 %), fever (7/14 patients, 50 %), emesis (7/14 patients, 50 %) and diarrhea (5/14 patients, 43 %). All AEs were mild and completely reversible. An infusion with paracetamol ended or eased the symptoms during the ertumaxomab administration immediately. There was no cardiotoxicity revealed by monitoring of the clinical heart function during treatment with ertumaxomab. All treatment-related adverse events that occurred in the total population are listed in detail in Table 3.

**Table 3:** Adverse events (AEs) with possible relationship to ertumaxomab treatment (Adverse Drug Reaction) graded according to CTC AE (version 4.0)

	Dose level 1		Dose level 5		Dose level 9		Dose level 13		Ʃ AEs
	(*n* = 3)		(*n* = 4)		(*n* = 3)		(*n* = 4)		(*n* = 14)
	*n*		*n*		*n*		*n*		*n* [%]
*AEs*	*G1/G2*	*G3*	*G1/G2*	*G3*	*G1/G2*	*G3*	*G1/G2*	*G3*	*Ʃ*
Fatigue	2	1	1	3	2	1	3	1	14 (100,0)
Cephalgia	2	-	3	-	3	-	4	-	12 (85,7)
Chills	3	-	2	-	3	-	3	-	11 (78,6)
Nausea	1	-	2	-	2	-	3	-	8 (61,5)
Emesis	1	-	2	-	2	-	2	-	7 (50,0)
Fever	1	-	2	1	2	-	1	1	7 (50,0)
Hypertension	3	-	2	-	1	-	1	-	7 (50,0)
Tumorpain	-	-	1	2	2	-	1	1	7 (50,0)
Pain^a^	1	-	4	-	1	-	-	-	6 (42,9)
Diarrhea	1	-	2	-	1	-	1	-	5 (42,9)
Pain in the limbs	-	-	2	-	-	-	3	-	5 (42,9)
↑CRP	1	-	1	-	1	-	1	-	4 (28,6)
Tachycardia	1	-	1	-	2	-	-	-	4 (28,6)
↑GGT	-	-	2	-	1	-	-	-	3 (21,4)
↑ALT/AST	-	-	1	-	1	-	-	-	2 (14,3)
↓Lymphocytes	-	-	2	-	-	-	-	-	2 (14,3)
Agitation	-	-	1	-	-	-	1	-	2 (14,3)
Dermatitis	-	-	2	-	-	-	-	-	2 (14,3)
Hypotension	1	-	-	-	1	-	-	-	2 (14,3)
Dizziness	-	-	-	-	1	-	1	-	2 (14,3)
Sensorium	-	-	2	-	-	-	-	-	2 (14,3
Allergic reaction^b^	-	-	1	-	-	-	-	-	1 (7,1)

Per patient every AE was counted once with its highest CTC grade. Only AE occurring in > 1 pt are listed (exception: allergic reaction)

*Abbreviations*: *G* grade according to CTC criteria, *CRP* C-reactive protein, *ALT/AST* aspartate aminotransferase/alanine aminotransferase, *GGT* gamma-glutamyl transpeptidase

^a^Pain: not further specified

^b^Symptoms: Sensorium, edema, tachypnea, cold hands, CRP elevation

### Efficacy

Three out of eleven patients evaluable for response showed disease stabilization or partial response after the first cycle of ertumaxomab (day 42–49): A partial response was seen in patient #6 (metastatic breast cancer) with a regression of hepatic metastases at first tumor evaluation. At the end of study, a progression in her axillary and hilar lymph nodes could be observed, but liver metastases were still in regress. Two patients (#1- rectal cancer and #2-head and neck cancer) were stable after the first cycle of ertumaxomab. They also had a progressive disease at end of study. There was no difference between tumor assessments according to RECIST. All three patients had Her2 IHC score 3+ tumors, two patients with disease stabilization were treated in dose level 1, one patient with partial response in dose level 5. Selected characteristics of disease together with an overall response of all patients treated with ertumaxomab are provided in Table 4.

**Table 4:** Corresponding study data and clinical charcteristics of patients

No.	Age range	Tumor	ECOG PS	No. of prior CHT	Organs involved	Her2 Status^1^	Dose level	No. of cycles	Best response
01	70–80	Rectum	1	4	LN, PUL, HEP, ADR	IHC 3+	1	2	SD
02	70–80	Head and neck	1	3	LN, PUL	IHC 3+	1	2	SD
03	40–50	Rectum	0	3	LN, SPLEEN, BLADDER, ABDOM WALL	IHC 3+	1	1	PD
04	60–70	Pancreas	1	3	ABDOM WALL	IHC 2+, ISH +	5	1	PD
05	50–60	Breast	0	4	PUL, OSS, LYMPHANG	IHC 3+	5	0	ND
06	50–60	Breast	0	3	HEP	IHC 3+	5	2	PR
07	50–60	Rectum	1	3	LN, PUL, PLEU, ABDOM WALL, PERI	IHC 2+, ISH+	5	1	PD
08	40–50	Stomach	0	4	LN	IHC 3+	9	1	PD
09	50–60	Breast	0	3	CHEST WALL, CEREB, HEP, OSS	IHC 3+	9	1	PD
10	50–60	Breast	0	4	LN, CUT	IHC 3+	9	1	PD
11	60–70	Head and neck	1	4	PUL, PLEU, OS	IHC 3+	13	1	PD
12	50–60	Stomach	0	4	PERI, OVAR	IHC 3+	13	1	PD
13	30–40	Breast	0	4	CUT	IHC 3+	13	1	PD
14	50–60	Stomach	1	1	LN	IHC 2+	13	1	PD

### Immunologic parameters

**Fig. 3:**
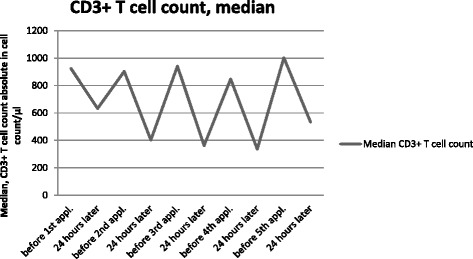
Median distribution of CD3+ T cells of all patients. Abbreviations: CD, Cluster of differentiation; appl, application

All patients showed a decrease of their CD3+ T cell count 24 h after receiving the investigational drug. One week later, their CD3+ lymphocyte count had normalized to baseline values. The decrease was dose dependent. Thus, the transient lymphocytopenia was more pronounced in higher levels of dose. The median distribution of the CD3+ T cell count of all patients is plotted in Fig. 3.

**Fig. 4:**
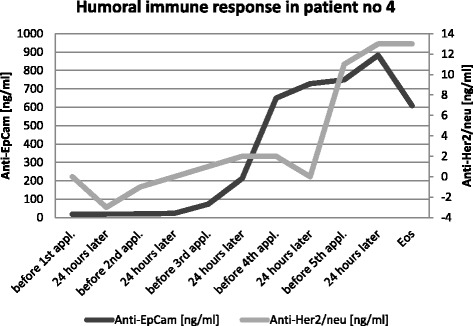
Humoral immune response against tumor-associated antigens (EpCam and Her2/neu) found in patient #4. Abbreviations: Appl, application

One out of five evaluable patients evolved anti-EpCam antibodies during the trial. The other four of these five patients had already preexistent anti-EpCam antibodies. The levels were in the range of 219–546 ng/ml and did not change significantly during treatment. Also, at end of study, anti-Her/neu antibodies were found in three out of five patients, all without pre-existing anti Her2/neu antibodies. Figure 4 shows a representative of a typical humoral immune response with corresponding, increasing levels for anti Her2/neu and anti-EpCam antibodies, which was seen in patient #4.

The analysis for HAMAs at screening showed that all tested patients were negative for HAMAs, whereas eight out of nine evaluable patients were found to be HAMA-positive at the end of study. There was no dose dependency seen for the development of HAMAs neither was a correlation to a different toxicity profile detected.

## Discussion

In this phase I trial we could show that treatment with ertumaxomab in a slow weekly escalating dosing regimen for five consecutive applications is feasible. The toxicity with this particular dosing schema is favorable. We observed no grade 4 toxicities and an acceptable incidence of grade 3 toxicities (fatigue in six, fever in two, and pain in three of 14 patients). Three patients had to be replaced as they received only two applications. Two of them had rapid tumor progression leading to cancer related death or liver failure. This relatively high replacement rate was mostly attributable to tolerant patient selection criteria as ECOG 2 patients were allowed to be enrolled. In comparison to published results from the previous phase I study with ertumaxomab in metastatic breast cancer [12], our study was associated with less acute toxicities, e.g. fever (all grades 50 % vs. 94 %). Furthermore, we did not observe any grade 4 ALT/AST (0 % vs. 42 %). These differences are most likely attributable to the different dosing and escalating schema. The study by Kiewe reported more cytokine release related symptoms, especially at the second and third application of the drug, which is related to the rapid escalation of the antibody dose. This issue is also reflected in the MTD achieved in the mentioned study, which was at 100 μg per single dose (in a 10-100-100 μg dosing schedule). In our study no DLT was observed in the main dosing cohorts and an MTD was not achieved. The results confirm the tolerability of the new schedule that allows the administrations of higher doses of the antibody. We planned to amend the protocol and further escalate the dose, when the trial withhold due to the unavailability of the study drug. Regarding the immune response to ertumaxomab, we made following observations: CD3+ T cell counts decreased 24 h post infusion, but normal values were obtained 1 week later. Transient lymphocytopenia was also observed in other clinical studies with trifunctional antibodies [12, 15] and is provoked by their mode of action: The full reversibility and the short recovery period indicate that the phenomenon is attributed to lymphocyte redistribution which could be confirmed in a pre-clinical model [17].

In contrast to the previous published study [12] of ertumaxomab with low incidence of HAMA (25 %) and HARA (31 %), we found HAMA development in eight of nine evaluable patients (89 %) in our study. Most probably, this may be referred to the prolonged dosing schedule used consisting of two cycles with each five applications. However, the induction of HAMA was not dose dependent, nor a correlation with a different toxicity profile was observed. In general, the development of HAMA was not of clinical and practical issue, indicating that a second treatment cycle could be safely administered. Remarkable in this context are results from a phase II/III study with the trifunctional antibody catumaxomab demonstrating a prolonged survival in HAMA positive compared to HAMA negative patients [18].

The monitoring of humoral responses to EpCAM and Her2/neu (in patients without pre-existing humoral immunity) showed increasing antibody titers during treatment in three of five evaluable patients, particular in one patient with corresponding increase in anti-EpCAM and anti-Her2/neu antibodies. Especially the observation of a humoral anti-EpCAM response confirms the potential of the trifunctional antibody to induce a polyclonal anti-tumor response against tumor-associated antigens which are not targeted by the trifunctional antibody itself. Similar immunological activity with increasing humoral and cellular immunity against different tumor-associated antigens was observed with the EpCAM-specific trifunctional antibody catumaxomab applied to gastric cancer patients in the adjuvant setting [19]. These results serve as a further indicator and proof of concept for the “trifunctionality” of ertumaxomab in vivo (with an involvement of antigen presenting cells), leading to immune recognition and priming of a polyclonal humoral immune response against tumor-associated antigens. However, due to the low evaluable patient numbers any conclusion between the induction of humoral immunity and clinical efficacy cannot be drawn.

Regarding the clinical efficacy, we observed two disease stabilizations (one patient with rectal cancer and one with head and neck cancer) and one partial remission (breast cancer) in 11 patients completing 1 cycle and being evaluable for response. The study was designed as a dose finding study. All patients were heavily pretreated, so responses are rarely expected in this patient group.

Although it is known that Her2 positive breast cancer benefits most from anti-Her2 strategies, our results support the evaluation of ertumaxomab in other Her2 positive malignancies as well.

Up to now, several anti Her2/neu strategies are adopted in breast cancer, the prototype disease for Her2/neu targeted therapy. The most known and most widely used is trastuzumab, a recombinant humanized monoclonal antibody against Her2/neu. Further approved drugs are lapatinib, a dual tyrosine kinase inhibitor of Her1 (also known as EGFR) and Her2/neu, trastuzumab-emtansine, an antibody-drug conjugate, consisting of trastuzumab and the cytotoxic agent mertansine (DM1), and pertuzumab, a Her2/neu dimerization inhibitor. The antibody based strategies are supposed to be functioning not only by blocking the signal transduction but also by an additional immunologic effect. Despite positive results from different phase I studies dealing with anti Her2/neu vaccination strategies, this concept of potential long lasting tumor control or even a disease eradication could not be established yet [20]. With the novel approach using the trifunctional antibody ertumaxomab different disadvantages and obstacles known from the classical vaccination concept could be overcome, like limitation of immune response to one or few epitopes, HLA restriction, or activation of only one pathway of immune response. Ertumaxomab is supposed to be acting by direct tumor cell/effector interaction and an indirect way by establishing a long lasting immune response.

Taken together, the results of our study have several implications:

First, we could show, that the dosing protocol consisting of two cycles with each five consecutive applications of ertumaxomab (50-100-150-200-300 μg) was feasible and tolerable. A further dose escalation to higher dose levels, as planned, is currently not possible as the company has to produce a new batch of the investigational drug. It has to be noted, that we could not yet define a MTD with our dosing schema.

Second, in diseases with approved anti Her2/neu drug therapies (breast cancer, gastric cancer), ertumaxomab could expand the therapeutic spectrum, either in (anti Her2) resistant/refractory disease or additionally to established anti Her2 strategies, or even adjuvant concepts.

Third, in Her2/neu overexpressing solid tumors, other than breast and gastric cancer, it could be evaluated as a new personalized treatment approach beyond standard therapy.

In conclusion, the results of our study together with previously published data warrant further evaluation of ertumaxomab in Her2/neu overexpressing solid tumors as a new targeted therapy adding to the armamentarium of personalized cancer treatment.

## Conclusion

In this study we could show that treatment with ertumaxomab in a slow weekly escalating dosing regimen for five consecutive applications (50-100-150-200-300 μg) is feasible and could confirm the tolerability of the new schedule that allows the administrations of higher doses of the antibody. The results of our study together with previously published data warrant further evaluation of ertumaxomab in Her2/neu overexpressing solid tumors.

## Abbreviations

AE, adverse events; ALT/AST, aspartate aminotransferase/alanine aminotransferase; CRP, C-reactive protein; CTC, common toxicity criteria; DLT, dose limiting toxicity; ECOG, Eastern Cooperative Group; EpCAM, epithelial cell adhesion molecule; Ertu, ertumaxomab; GGT, gamma-glutamyl transpeptidase; HAMAs, human anti-murine antibodies; Her2/neu, human epidermal growth factor receptor 2; IHC, immunohistochemistry; ISH, in-situ hybridization; LVEF, left ventricular ejection fraction; MTD, maximum tolerated dose; pts, patients; RD, recommended dose; RECIST, response evaluation criteria in solid tumors; SAEs, serious adverse events; SAR, serious adverse reaction; TAA, tumor-associated antigen
